# tVNS and obesity-related inflammation: a prospective sham-controlled randomized crossover study protocol

**DOI:** 10.1186/s40359-026-05219-5

**Published:** 2026-08-04

**Authors:** Luisa Kaluza, Anne Kühnel, Ernesto Picòn-Galindo, Mona Hergenröther, Hesham Alkhateeb, Simon Michalski, Dalila Juliana Silva Ribeiro, Susanne V. Schmidt, Dagmar Wachten, Nils B. Kroemer

**Affiliations:** 1 Department of Psychiatry and Psychotherapy, Section of Medical Psychology, University Hospital Bonn, University of Bonn, Venusberg Campus 1, Bonn, 53127 Germany; 2 Department of Psychiatry and Psychotherapy, Tübingen Center for Mental Health, University of Tübingen, Tübingen, Germany; 3 German Center for Mental Health (DZPG), partner site Tübingen, Tübingen, Germany; 4 German Center for Diabetes Research (DZD), Neuherberg, Germany; 5 Department of Neuroimmunology and Neuromuscular Diseases, Centre for Neurology, University Hospital Bonn, University of Bonn, Bonn, Germany; 6 Institute of Innate Immunity, Biophysical Imaging, University Hospital Bonn, University of Bonn, Bonn, Germany; 7 Institute of Clinical Chemistry and Clinical Pharmacology, University Hospital Bonn, Bonn, 53127 Germany

**Keywords:** Overweight, Metaflammation, Effort, Motivation, Vagus nerve stimulation

## Abstract

**Background:**

Overweight and obesity are characterized by detrimental effects on energy metabolism and an increase in adipose tissue, leading to chronic low-grade inflammation. This metaflammation may contribute to mental symptoms, such as reduced mood and motivation. Inflammatory processes are modulated by the cholinergic anti-inflammatory pathway (CAIP) that can be targeted with vagus nerve stimulation (VNS). However, the effects of non-invasive transcutaneous VNS (tVNS) on inflammation have predominantly been investigated in small feasibility studies in humans. Whereas acute anti-inflammatory effects of tVNS have been reported, no study has investigated the effect of daily home-based application on obesity-related metaflammation to date.

**Methods:**

To assess tVNS-induced effects on inflammation, motivation, and mood, we are conducting a single-blind, randomized crossover study in participants with an elevated BMI (target *N* = 60, 27–35 kg/m², 18–40 y). Participants will self-administer high- (active) and low-intensity (sham) stimulation daily (≥ 30 min) for ~ 14 days each (≥ 7 d washout). We will assess peripheral (cytokine levels, circulating immune cells) and central (diffusion weighted imaging) inflammation, motivation (using an effort allocation task during functional MRI), and mood at baseline and after each stimulation phase. During each stimulation phase, participants will complete daily ecological momentary assessments of mood and food choices. We will use mixed-effects models to evaluate differences in slopes of high- vs. low-intensity tVNS.

**Discussion:**

This study uses a 14-day home-based tVNS protocol (≥ 30 min daily) accompanied by daily ecologocial momentary assessments to investigate mid-term effects on chronic low-grade inflammation, mood, and motivation. The multimodal study design including a comprehensive panel of peripheral and central inflammatory markers enables the integration of inflammatory and behavioral outcomes and will provide important evidence regarding the efficacy of tVNS in chronic inflammation, based on a study with sufficient statistical power. In addition, the study could provide pathophysiological insights into the link between obesity and symptoms of depression via inflammatory processes. Ultimately, the study will facilitate future research on vagus nerve stimulation to complement the current set of therapies in overweight and obesity.

**Trial registration:**

The study has been preregistered at ClinicalTrials.gov (https://clinicaltrials.gov/study/NCT06954844) on March 21, 2025, Version 1.

**Supplementary Information:**

The online version contains supplementary material available at 10.1186/s40359-026-05219-5.

## Background

Obesity is characterized by chronic low-grade peripheral inflammation (metaflammation). Several studies have shown elevated cytokine levels and alterations in circulating immune cells in individuals with overweight or obesity [2, 8, 16, 54, 77, 94]. In addition, obesity is associated with central inflammation, such as increased cellularity and edema [22, 79, 80]. Beyond the well-established impact of obesity on metabolic and cardiovascular function [99], obesity is often also comorbid with mental disorders, such as depression [20, 40]. Emerging evidence supports a mechanistic role for inflammation in driving mood and motivational disturbances. In animal [30, 32, 33] and human studies [13, 14, 23, 33], inflammatory stimuli induce depressive-like behavior and alter dopamine or reward-related signaling in the brain. Higher levels of inflammatory markers have also been linked to reduced functional connectivity within the cortico-striatal motivational circuit in patients with depression, which was associated with higher anhedonia and psychomotor slowing [31]. Likewise, higher inflammation has been linked to altered reward anticipation [11] and elevated central inflammation has been found in the dopaminergic midbrain of patients with depression compared to healthy control participants [51]. Similar to the effects of inflammation, diet-induced obesity is also associated with altered dopamine transmission, reward deficiency, and depressive behavior both in animals [38, 42, 48, 83] and humans [20, 40, 43, 87]. Recent evidence has associated low-grade inflammation in obesity with reduced overall motivation to expend effort for rewards [18]. Taken together, this indicates that obesity-associated inflammation may mechanistically contribute to the higher incidence of motivational and mood disturbances in people with obesity. This positions overweight and obesity as a potential model condition for anti-inflammatory interventions.

While Glucagon-like Peptide-1 (GLP-1) receptor agonists (RA) have led to striking improvements in weight reduction interventions [98], there remains an unmet need for alternative treatments in individuals with overweight or class-I obesity due to potential negative health effects, including inflammation and mental symptoms. This applies to both at-risk individuals before they are eligible for GLP-1 RAs or during a maintenance phase after initial weight loss. To counteract inflammatory effects, neuromodulation of the cholinergic anti-inflammatory pathway (CAIP, [10]) via electrical vagus nerve stimulation (VNS) has emerged as a promising approach. Recently, invasive VNS has received FDA approval for the treatment of rheumatoid arthritis [91]. In addition, prolonged VNS is effective in treatment-resistant depression [1, 76, 78], although it is not clear whether this is due to anti-inflammatory effects. Transcutaneous VNS (tVNS) provides a non-invasive and well-tolerated alternative to invasive VNS [28, 29, 75]. Acute tVNS has been demonstrated to improve mood recovery [35] and effort invigoration [34, 69]. tVNS has been shown to successfully activate the nucleus tractus solitarii, the primary target of vagal afferents in the brain, in humans [9, 36, 89, 102]. In addition, tVNS elicits efferent effects on peripheral organs, as indexed by changes in gastric myoelectric activity [46, 90], suggesting that CAIP could be activated. Accordingly, tVNS reduces inflammation in rodents when administered during acute immune challenges [84] and during chronic inflammation [6]. In humans, tVNS has been shown to reduce inflammation mainly in acute inflammation [58, 101, 103], while findings for chronic inflammation are inconsistent [7, 70, 85, 86]. The current evidence might be limited due to small sample sizes, variable stimulation settings [82], and a lack of comprehensive inflammatory phenotyping. Sufficiently powered studies investigating the prolonged effects of daily conventional auricular tVNS administration on chronic low-grade peripheral and central inflammation, such as in people with overweight/obesity, are lacking.

To address this translational gap, this study uses prolonged (≥ 30 min of daily stimulation over ~ 14 days), self-administered tVNS with high-intensity stimulation as active condition and low-intensity stimulation as a control condition in 60 participants with overweight or class-I obesity. We will evaluate midterm effects on both peripheral and central inflammation, as well as on mood and motivation, while recording and monitoring potential side effects of the intervention. By simultaneously assessing inflammatory markers and behavioral outcome measures, the study investigates potential therapeutic effects of tVNS and provides insights into the pathomechanisms linking obesity-related inflammation with disturbances in mood and motivation.

## Methods

### Participants

We aim to include 60 participants aged 18 to 40 years with an elevated BMI (27–35 kg/m²), a range selected to reflect a population at increased risk for long-term consequences of obesity, including metaflammation. We will compensate for dropouts until the planned sample size is reached and 60 participants have completed all sessions. The sample size was determined to detect small-to-medium within-subjects effects (*d*_*z*_ ~0.40) with a statistical power of 1-*β* = 0.86, consistent with previous findings [69, 89]. Prior to participation, all individuals provide written informed consent after being given sufficient time for consideration and the opportunity to ask questions to the study team. Optionally, participants may consent to providing additional blood samples for the Mental Health Biobank Bonn using a separate consent form. Specific participant insurance is not provided; liability is only covered in cases of proven institutional negligence. The study has been preregistered at ClinicalTrials.gov (https://clinicaltrials.gov/study/NCT06954844) on March 21, 2025 and a statistical analysis plan has been uploaded to OSF. The first participant started on the 8th of April in 2025, and at the time of submission of this study protocol in July 2026, 27 participants had completed the study. Recruitment is expected to be completed in May 2027.

### Screening procedure

Participants are recruited through local flyers, distributed in public places, such as libraries and cafeterias, and in settings frequented by at-risk individuals, such as weight management groups. In addition, we use social media posts to recruit participants. Prior to enrollment, we conduct phone screenings with potential participants to determine eligibility. Candidates are excluded if they have a current or past diagnosis of brain injury/surgery or neurological condition with permanent effects, epilepsy, stroke, schizophrenia, bipolar disorder, severe substance use disorder, heart disease that precludes use of tVNS, diabetes (type 1 or 2), or chronic inflammatory diseases (e.g., rheumatoid arthritis, Crohn’s disease, etc.). Moreover, we exclude participants who took medication or received electroconvulsive therapy to treat a mental, metabolic, or neurological disorder (e.g., selective serotonin reuptake inhibitors, GLP-1 RA) or took anti-inflammatory medication in the 3 months prior to the start of the experiment. However, hormone treatments that normalize function (e.g., L-thyroxin) and the occasional use of painkillers (e.g., ibuprofen) are not excluded. Additionally, individuals are excluded if they fulfilled the criteria for an eating disorder, obsessive-compulsive disorder, or somatic symptom disorder within 12 months before the experiment. We do not include pregnant or breastfeeding women, people with an elevated BMI due to high fat-free mass (e.g., athletes), or people with considerable weight change (> 10%) within the last 6 months before the experiment (based on the phone screening). In addition, contraindications for MRI (e.g., metal implants) or tVNS application (e.g., sore or diseased skin areas on the outer right ear, cerebral shunt) preclude inclusion in the study.

### Experimental procedure

**Fig. 1:**
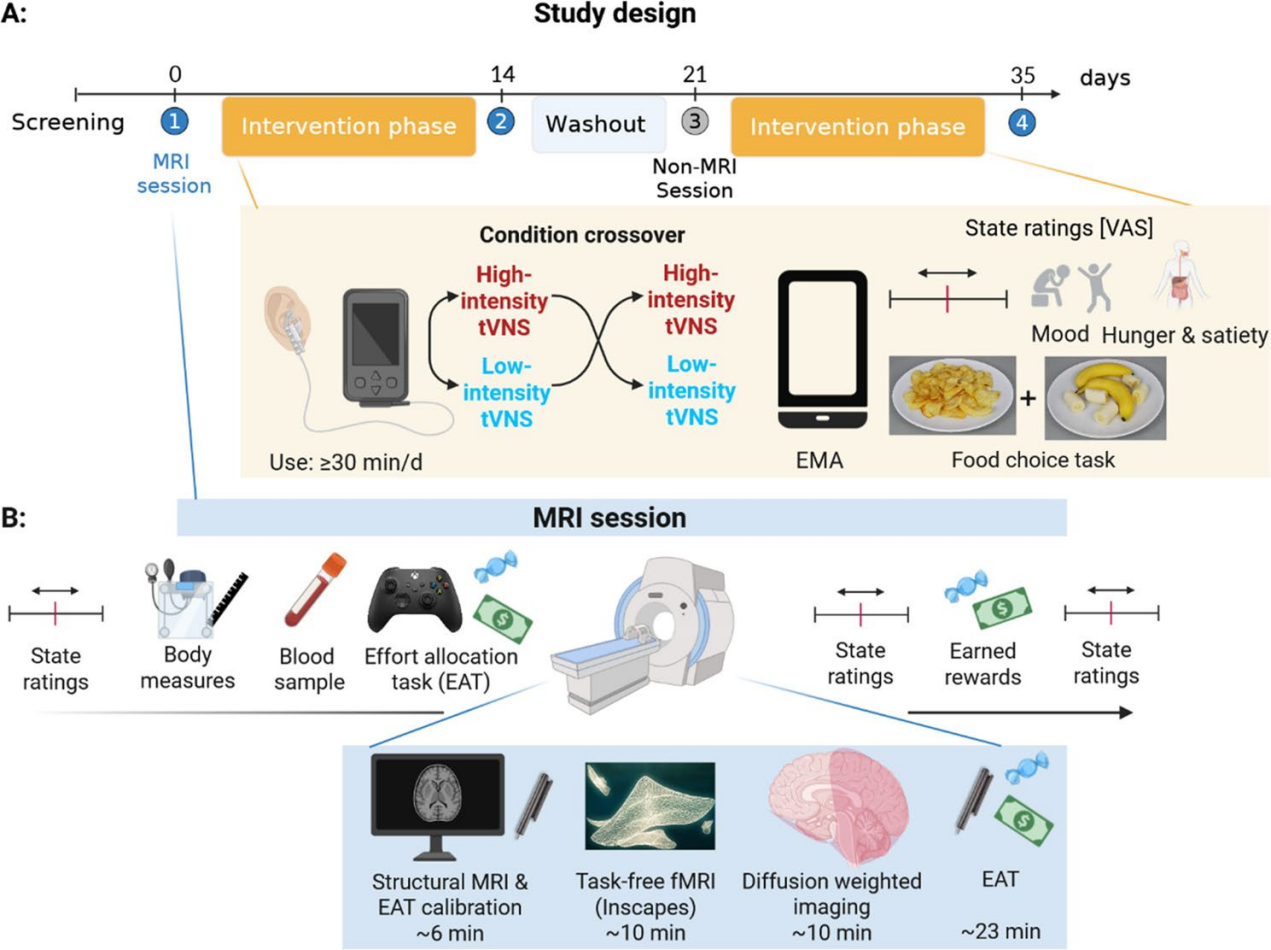
Overview of the experimental setup. **A**: The study uses a single-blind randomized crossover design with four sessions and two intervention phases, separated by a brief washout phase. Three sessions include an MRI assessment. During the third session, we assess mood, body measures and fasting blood parameters. During the intervention phase, participants are instructed to stimulate the vagus nerve with high or low intensity for at least 30 min each day and to complete ecological momentary assessment (EMA) including a food choice task. **B**: Each MRI session follows a standardized procedure to assess mood (state ratings), inflammation (peripheral: blood sample, central: diffusion weighted imaging), and motivation. VAS = visual analog scale

Participants receive high-intensity or low-intensity tVNS at the cymba concha of the right ear in a single-blind, randomized, crossover design. The randomization (high- or low-intensity stimulation first) list was created prior to study initiation using custom MATLAB code (randperm function) by the study team. The study team adds participants to the list in the order of inclusion based on the assigned ID. To maintain allocation concealment, condition assignments are disclosed only during the lab session. Participants are instructed to apply stimulation at home for at least 30 min and up to 4 h daily over a period of about 14 days for each condition. Both conditions are separated by a washout period of ≥ 7 days. The low-intensity stimulation serves as control condition because it is unlikely to activate vagal afferents. To improve blinding, we instruct participants that we compare the high-intensity stimulation to a low-intensity stimulation that is deliberately below the sensation threshold. Throughout the study, participants are invited to 4 lab-based sessions at the Section of Medical Psychology, Department of Psychiatry & Psychotherapy, University Hospital Bonn, Germany (see Fig. 1A).

**Fig. 2:**
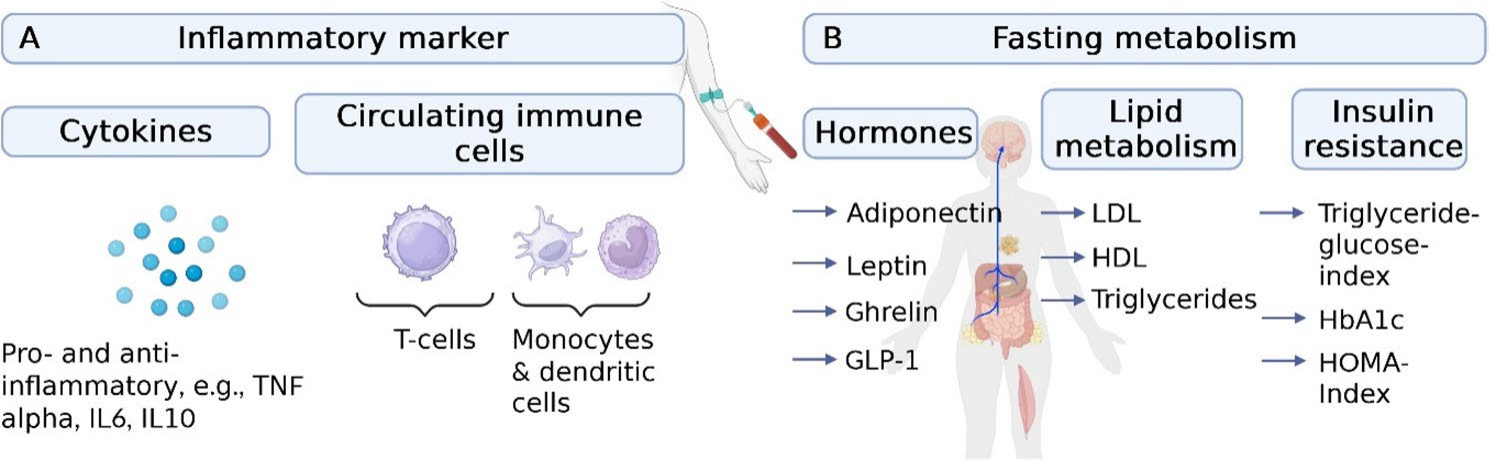
Overview of the blood parameters of Session 1, 2, and 4. **A**: Peripheral inflammation is measured via levels of pro– and anti-inflammatory cytokines and other biomarkers of inflammation using highly sensitive immunoassays. The specific panel will be selected after data collection is complete and before analysis begins. In addition, we will evaluate circulating immune cells by analyzing peripheral blood mononuclear cells. **B**: Fasting metabolism is evaluated by hormone levels (adiponectin, leptin, ghrelin, glucagon-like peptide-1 (GLP-1)), lipid metabolism (triglycerides, high-density lipoprotein (HDL), low-density lipoprotein (LDL)), and measures of insulin resistance (Homeostasis Model Assessment (HOMA-Index), triglyceride-glucose-index, HbA1c)

For all sessions, we instruct participants to come in an overnight fasted state (≥ 12 h). Each MRI session follows the same standardized procedure (Fig. 1B): At the beginning of the session, participants report their current metabolic and mood state using a visual analog scale (VAS) [26, 35]. Then we assess body measures (BMI, waist-to-hip ratio, percentage of body fat, resting heart rate, and blood pressure) and take a blood sample to measure inflammation (cytokines, circulating immune cells) and fasting metabolism (for an overview of blood parameters, see Fig. 2). During the first session, participants then complete a food cue reactivity task [52, 66], rating the liking, wanting, healthiness, and environmental sustainability of 60 food items. This information is used for the food choice task during the ecological momentary assessment (EMA) phase. Participants then complete a short version of the effort allocation task (EAT) using a gamepad to quantify motivation and collect ratings of wanting and exertion [34, 69]. After the behavioral EAT, participants enter the MRI for the neuroimaging part of the session. First, we acquire a field map and a T1 weighted (T1w) anatomical image. During these scans, we calibrate the grip force device used in the MRI version of the EAT. Second, we acquire a 10 min resting state fMRI measurement with the Inscapes video [67, 89, 93]. To measure central inflammation, we then acquire a diffusion weighted imaging sequence [50, 79, 80, 95]. Afterwards, participants perform the rapEAT (~ 23 min), which is an adapted version of the EAT for neuroimaging. After neuroimaging, participants complete state VAS a second time. We then pay out the rewards earned in the EAT (80% of the calories as breakfast and 20% of the calories as snacks [69]. Afterwards, participants complete state VAS a third time.

In the first session, participants currently or formerly diagnosed with depression undergo a structured clinical interview to assess atypical symptoms of depression [100]. We instruct the participants on how to use the tVNS device, which is either set to 0.1 mA (low-intensity stimulation) or individually calibrated and then set to a level corresponding to mild pricking (high-intensity stimulation). During the following intervention phase (~ 14 days), stimulation usage will be logged by the device. In addition to the stimulation, participants are asked to complete EMA once a day to report their metabolic and mood states [49, 68]. After the state ratings, they complete a food choice task to track potential changes in dietary choices. At the beginning of the second session, we ask participants whether they experienced any potential side effects and how they perceived the stimulation. This is used to determine the effectiveness of blinding and replaces the food cue reactivity task. Except for these differences, the second session at the end of the first intervention phase follows the same procedure.

After the washout period, an adapted session without neuroimaging takes place. First, we take a selection of the previously assessed anthropometric measures (BMI, waist-to-hip ratio, resting heart rate, and blood pressure) and fasting blood samples (glucose, insulin, triglycerides, high-density lipoprotein (HDL), low-density lipoprotein (LDL), HbA1c, and a standard differential blood count). In addition, participants complete two state VAS (at the beginning and end of the session). Participants receive the tVNS device configured for the second crossover condition and complete the intervention phase again for ~ 14 days. The study concludes with Session 4 analogous to Session 2. In addition to EAT earnings, participants receive either 80 € or partial course credit as compensation.

### Participant and public involvement

The results will be disseminated through open access publications as preprints and then submitted to peer-reviewed journals. This will be complemented by public outreach activities (e.g., press releases and social media posts). We developed the research question and outcomes without participant or public involvement.

### Intervention: transcutaneous Vagus Nerve Stimulation (tVNS)

All participants are instructed to stimulate the right cymba concha, a region that is innervated by the auricular branch of the vagus nerve [36]. We use tVNS R (tVNS technology, Erlangen, Germany) that enables the adjustment of stimulation parameters and logging of use outside the lab. For the high-intensity stimulation, the intensity is set to an individually calibrated amplitude corresponding to a mild pricking sensation (using a staircase procedure). We use the conventional stimulation protocol (30 s ON, 30 s OFF, 25 Hz frequency, 250 µs pulse width) [34, 69]. If participants report a painful stimulation after the initial calibration, we will lower the stimulation intensity in steps of 0.1 mA, until the stimulation corresponds to mild pricking again. For the low-intensity stimulation, the amplitude is set to the minimum of 0.1 mA (1 s ON, 30 s OFF, 1 Hz frequency, 250 µs pulse width). This amplitude is too low to activate vagal afferent fibers [29]. Participants will be unblinded and excluded if continued tVNS may pose a health risk to them (e.g., due to changes in criteria that preclude safe use).

### Tasks

#### Effort Allocation Task (EAT)

**Fig. 3:**
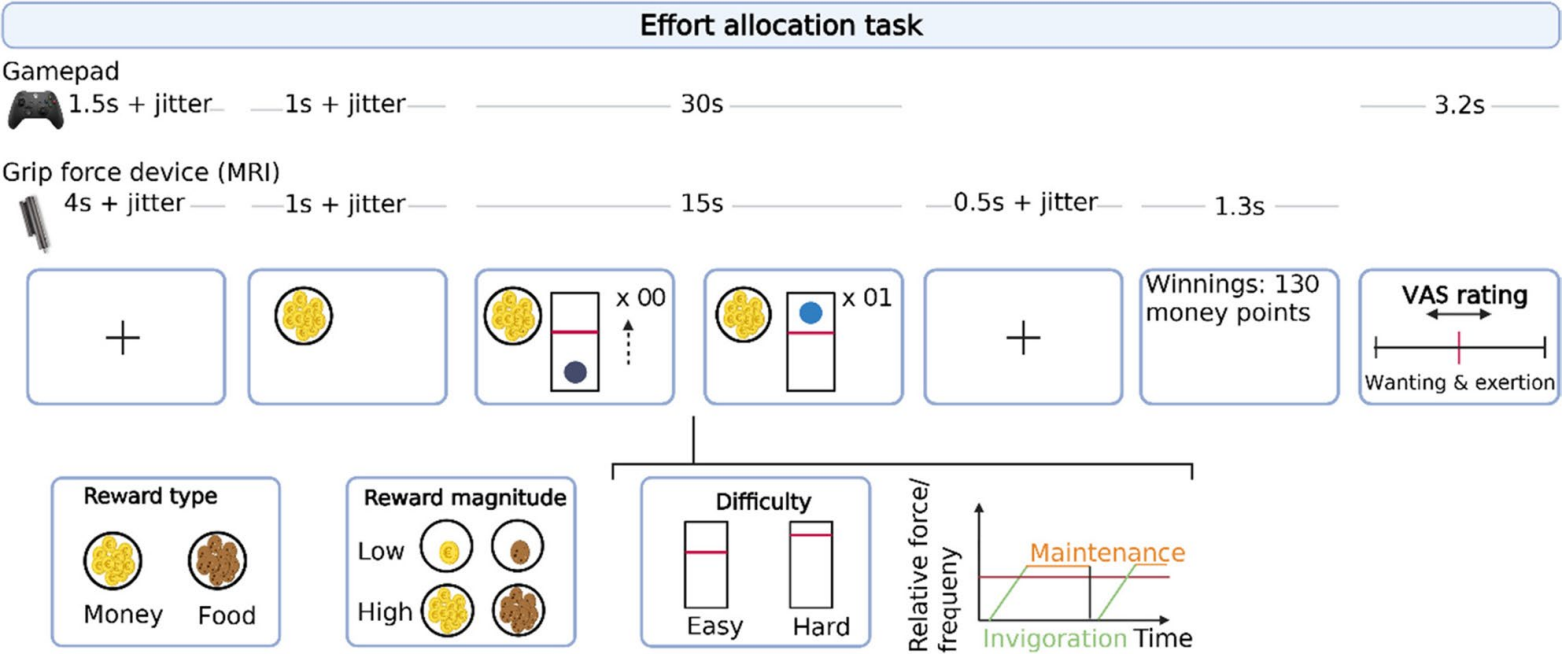
Schematic illustration of the effort allocation task. During the effort allocation task, participants move a ball above a red difficulty line by either using a gamepad (button press frequency) or a grip force handle to collect reward points. The difficulty levels are calculated according to the individual’s maximum frequency/force determined before the task in the first session (frequency) or in each session (force). The calculated effort is tracked over time to estimate complementary motivational indices (invigoration and effort maintenance). To measure adjustments of motivation due to costs and benefits, the task includes manipulations in reward type, magnitude, and difficulty. In addition, when using the gamepad, participants also rate their wanting and exertion after each trial using a visual analog scale (VAS) ranging from 0 (lowest rating) to 100 (highest rating)

To measure motivation, we use a validated effort-based cost-benefit paradigm that we previously adapted [34, 69] from Meyniel et al., [65]. In the EAT, participants are asked to move a ball in a tube above a red difficulty line by exerting effort (i.e., by rapidly pressing a button on the gamepad or squeezing a grip force handle inside the scanner) to collect reward points. Trials within the task vary in difficulty (easy vs. hard), reward type (food vs. money), and reward magnitude (1 point vs. 10 points for every second that the ball remains above the line). Participants receive their total earnings after the MRI for both versions (100 points = 0.25 € | 30 kcal). The task will be used to assess invigoration (i.e., the slope of the initial approach reflecting behavioral drive) and effort maintenance (i.e., average relative force/frequency in the trial; Fig. 3; https://github.com/neuromadlab/Tasks/tree/master/Effort_Allocation_Task/Project_Versions/BON006_tVNS_inflammation [34, 69]. The behavioral EAT uses 75% or 85% of the individual’s maximum frequency as difficulty levels (counterbalanced throughout the task, 3 repetitions of all combinations, 24 trials in total, ~ 16 min). For the rapEAT during neuroimaging [55, 71], the difficulty levels are 65% or 75% of the individual’s maximum force (counterbalanced throughout the task, 6 repetitions of all combinations, 48 trials). For the grip force version, we assess the individual’s maximum force for each MRI session separately since the device requires recalibration.

#### State ratings (visual analog scales)

The state rating task uses 24 questions to assess metabolic and mood states on a VAS ranging from 0 (lowest rating) to 100 (highest rating) (https://github.com/neuromadlab/Tasks/tree/master/VAS/Project_Versions/BON006_tVNS_inflammation; [35]). The four items assessing metabolic states are: hungry, thirsty, sated, and tired. For mood, we use the Positive and Negative Affect Schedule (PANAS) items [96]. Questions are presented on a computer screen, and participants answer using the computer mouse.

#### Food cue reactivity task

The task is an adaption from the previously described food cue reactivity task (https://github.com/neuromadlab/Tasks/tree/master/Food_Cue_Reactivity_Task/Project_Versions/BON006_tVNS_inflammation; [52, 66]: In this task, we use a subset of 60 food pictures selected from standardized food pictures [17] to cover a wide range of macronutrient compositions [92]. In addition to the previously used liking (vertical scale) and wanting (horizontal scale), participants are asked to rate food pictures regarding healthiness and environmental sustainability (horizontal scales ranging from 0 (not at all) to 100 (very). The term ‘environmental sustainability’ was chosen as it has been established in prior survey research [97], thereby minimizing ambiguity in participants’ understanding of the concept. Each image is shown twice and then followed by two of the four rating scales. Scale combinations and image order are randomized and there is no time limit per rating. At the end of the task, participants are asked to order the four rating domains according to their personal relevance.

#### Ecological momentary assessment

Participants complete daily EMA during each intervention phase to repeatedly assess state ratings and changes in food choices using the online platform Pavlovia (pavlovia.org, https://github.com/neuromadlab/Tasks/tree/master/Food_Cue_Reactivity_Task/Project_Versions/BON006_tVNS_inflammation). The task input and answers to state questions can be given on a smartphone, tablet, or computer, using a mouse or touch inputs. In addition to previously used items [49, 68], such as metabolic (e.g., hunger, satiety), and mood-related (e.g., happiness, sadness) states, we collect related items on mental state (motivated and lonely), and subjective control over food (three items) using VAS (0-100). We also assess whether participants have stimulated the vagus nerve on that day, whether they took pain killer on that day [binary yes or no answers], their physical activity: minutes of moderate or strenuous physical activity today [< 5, 5–14, 15–29, 30–44, 45–59, 60–89, ≥ 90], food consumption: amount of food consumption in comparison to last four weeks [less, same, more], and interoception: awareness of the location of tension in the body [6 point Likert scale; never to always], awareness of feelings in the belly [6 point Likert scale; strongly disagree to strongly agree].

After answering the state questions, participants perform a food choice task. It will be used to assess changes in participants’ preferences based on the ratings provided in the first session. Therefore, 20 pictures with moderate liking ratings above 0 (i.e., not disliked) are selected and presented in 30 choice trials (4s to respond, ~ 150s) between two options. Each image is shown three times and paired randomly with a second image out of the selected 20 pictures (no repetition of combinations).

### Data acquisition

#### Blood parameter

To assess peripheral inflammation and fasting metabolism, blood samples are collected at the beginning of each session. Glucose, insulin, triglycerides, HDL, LDL, HbA1c, and differential blood counts are analyzed by the Central Laboratory of the University Hospital Bonn. EDTA plasma (untreated and treated with aprotinin (500 KIU/ml)) will be immediately processed after the blood draw (centrifugation settings: 4 °C, 10 min, 2000 g) and then stored in aliquots at -80 °C. These plasma aliquots will be used to analyze additional hormones with ELISA kits, and to assess plasma levels of pro- and anti-inflammatory cytokines and other biomarkers of inflammation using highly sensitive immunoassays (e.g., NULISA).

In addition to the standard differential blood count, we will evaluate circulating immune cells by analyzing peripheral blood mononuclear cells (PBMC) that are isolated from fresh human peripheral blood by density gradient separation using Ficoll™ Paque Plus (cytiva, Sweden). In brief, whole blood is collected into EDTA-containing tubes (SARSTEDT, Germany). Blood is diluted 1:1 with Dulbecco’s Phosphate Buffered Saline (DPBS; Thermo Fisher Scientific, USA) and carefully layered onto 15 mL of Ficoll™ Paque Plus. Samples are centrifuged (700 g, 20 min, room temperature) and the PBMC layer is collected and washed using DPBS. Residual erythrocytes are removed by incubating the sample 3 min in red blood cell lysis buffer (BioLegend, USA). Afterwards, the cells are washed again using DPBS. Cell counts and viability are determined with 0.4% Trypan Blue Stain (Thermo Fisher Scientific, USA) using the automated TC20™ Cell Counter (Bio-Rad, USA), ensuring high cell viability for subsequent experiments. To quantify circulating immune cells from PBMCs, we will assess immune cells in three different panels (see Table 1, for an overview of all antibodies and gating strategies, see SI). Briefly, we incubate the cells for 10 min at room temperature with human FcR Blocking Reagent (Miltenyi, Netherlands) to block unspecific binding sites. Afterwards, cells are incubated with the corresponding antibodies (4 °C, 30 min, light protected). Samples are washed twice with staining buffer solution (1 x PBS supplemented with 2 mM EDTA and 0.5% BSA) and finally the cells of each panel are resuspended in 200 µL of staining buffer solution. Stained samples are acquired on a flow cytometer (Attune NxT, Thermo Fisher). Data will be analyzed using FlowJo (BD Biosciences) and R [73, 74].

**Table 1:** circulating cells investigated using flow cytometry

T-cell panel 1	T-cell panel 2	Monocytes and dendritic cells
CD4 + Tconv	CD4 + T-helper cells	Dendritic cells
Naïve	Th1	cDC1
Central memory	Th1.17	cDC2
Effector memory	Th2	pDC
TEMRA	Th17	
CD8 + T-cells		Monocytes
Naïve		Classical
Central memory		Non-classical
Effector memory		Intermediate
TEMRA		
CD4 + Treg		
Activated		
Resting		

*Tconv* conventional T-cells, *TEMRA* terminal effector memory re-expressing CD45RA, *Treg* regulatory T cells, *cDC* conventional dendritic cells, *pDC* plasmacytoid dendritic cells

#### Diffusion weighted imaging acquisition and preprocessing

To assess central inflammatory responses to tVNS, we acquire one planar AP diffusion weighted imaging (DWI) sequence (voxel dimensions: 2 × 2 × 2 mm, TE = 95 ms, TR = 10,000 ms, transverse orientation, flip angle = 90°, 26 directions, b-values ranging from 0 to 1400 s/mm^2^) and two non-diffusion images (b-value = 0, one in AP and one in PA direction, respectively) per participant and MRI session using a 3 T Siemens scanner (Magnetom Trio) with a 32-channel head coil. Data will be preprocessed using the FSL [47] command *fslmerge*, *eddy correct* and *topup* [3], and the diffusion basis spectrum imaging (DBSI) Toolbox (MATLAB code) kindly provided by Sheng-Kwei Song [95]. Thereby, we will calculate proxies for tissue edema (isotropic diffusion (*f*(D), D = 0.3–3.0 µm^2^/ms), cellularity (isotropic diffusion (*f*(D), D ≤ 0.3 µm^2^/ms), and dendrite/axonal density (anisotropic diffusion) as readouts of central inflammation.

#### fMRI data acquisition and preprocessing

fMRI images are collected at rest (10 min) and during the EAT. Task fMRI will be used to measure brain responses related to reward, effort, and invigoration. All fMRI data will be used to investigate functional connectivity. We acquire T2*-weighted echo-planar imaging (EPI) sequences (40 ascending axial slices to cover the whole brain, TR = 2.66s, TE = 30ms, flip angle = 77°, matrix size = 64 × 64, field of view = 192 × 192 mm², and voxel size = 3 × 3 × 3 mm³). To control for inhomogeneity of the magnetic field, we acquire a field map, Siemens gradient echo field map sequence: short TE = 4.92ms, long TE = 7.38ms. For anatomical co-registration, a structural T1w image is collected at the beginning of each session (208 sagittal slices, flip angle = 9°, matrix size = 320 × 320 and voxel size = 0.8 × 0.8 × 0.8 mm³). Imaging data will be preprocessed using fMRIprep [24]. Smoothing ([8 8 8] mm) and first-level statistics will be implemented in SPM, version 12 (Wellcome Department of Imaging Neuroscience, University College London, London, UK [39]). Regions of interest will be defined by an extended version of the Harvard-Oxford brain atlas [21, 72, 89]. The following regressors will be included in the default first-level models: anticipatory phase (onset when reward cues are shown, duration = 0s), the work phase (duration = 15s), and feedback presentation (duration = 0s). The cue regressor is modeled separately for food and money trials and both include a parametric modulation by reward magnitude (low, high). The regressor for the work phase includes a parametric modulator for difficulty (low, high) and the feedback regressor parametric modulations for reward magnitude and difficulty. Moreover, we will include regressors capturing the exerted effort at that time: force/TR and the change in force from timepoint to timepoint: delta(force)/TR. To test the effects of tVNS in pre-specified regions of interest, we will extract average contrast estimates from the individual images for each session and analyze them using linear mixed-effects models. In addition, we will explore voxel-wise whole-brain effects using second-level full factorial models in SPM. The main contrasts of interest are reward magnitude effect for cue anticipation across food and money trials and delta(force)/TR to capture invigoration.

#### Self-reported questionnaires

Participants are asked to fill out self-reported questionnaires to characterize the sample at baseline and to track additional symptoms over the course of the study. To assess eating behavior at baseline, we use the Eating Disorder Examination Questionnaire [27], the Salzburg Emotional Eating Scales [63], the Salzburg Stress Eating Scales [64], the Three-Factor Eating Questionnaire [88], and the Food Cravings Questionnaire-Trait-reduced [62]. In addition, we collect questionnaires on substance use (Alcohol Use Disorder Identification Test [81], Cannabis Use Disorders Identification Test [5], Fagerstrom Test for Nicotine Dependence [25]). Moreover, participants report somatic symptoms using the Patient Health Questionnaire-15 [53], anxiety using the Liebowitz Social Anxiety Scale [60] and State-Trait Anxiety Inventory-Trait [59], and trait motivation using Behavioral Inhibition System/Behavioral Approach System scales [15].

Over the course of the study (i.e., at baseline and after 14 days of each stimulation), we assess symptoms of depression (Beck Depression Inventory II, BDI-II [45]), anhedonia (Snaith-Hamilton Pleasure Scale in German, SHAPS-D [37]), and apathy (Apathy Motivation Index, AMI [4]). In addition, we assess physical activity using the International Physical Activity Questionnaire [19] and interoception using the Multidimensional Assessment of Interoceptive Awareness (v2) [61] and the Visceral Sensitivity Index [57] questionnaire. We assess eating behavior using a food frequency questionnaire (DEGS, [44], the Yale Food Addiction Scale [41], and the Power of Food Scale [12]. We also systematically record potential tVNS side effects, participants’ expectations regarding tVNS, and blinding.

#### Data management and quality assurance

Data on behavioral tasks and on self-reported questionnaires are collected using electronic data capture systems. All data are then securely transferred to institutional servers. All personal data is password-protected and all data are acquired and stored in pseudonymized form until data collection has been completed. After study completion, all data will be anonymized. To ensure consistency and reliability across sessions, data acquisition follows standardized protocols including instructions on tVNS usage and calibration. Blood sampling and the MRI acquisition is performed by trained personnel with medical or psychological background. To enhance transparency, reproducibility, and adherence to open science principles, open-access analysis scripts of the EAT are available via GitHub (https://github.com/neuromadlab/Tasks/tree/master/Effort_Allocation_Task/Analyses/general). Using customized MATLAB scripts, the study team monitors adherence to stimulation protocols, completion of EMA, questionnaires and assessment of primary outcomes. As the study poses minimal risks and involves a short intervention period, safety is monitored by the study investigators in accordance with ethical oversight requirements. No further outcome data will be collected for participants who have discontinued. No formal auditing is planned. Any substantial changes to the study protocol will be communicated to the ethics committee and trial registries as appropriate.

#### Participant retention and follow-up

To improve compliance with the study protocol, participants receive a completion bonus as monetary compensation. In addition, participants also receive daily reminders to complete EMA and perform the tVNS stimulation. Participants are also informed that the tVNS devices record their use.

### Statistical analyses

#### Baseline characteristics and group comparisons

Descriptive statistics will be reported for age, sex, BMI, waist-to-hip ratio, use pattern of tVNS (e.g., frequency, duration; split by high and low-intensity stimulation), and depressive symptoms (BDI-II). Statistical analyses will be reported using summary tables and figures. Continuous variables will be summarized with means and standard deviations. Categorical variables will be summarized by counts and by percentage of participants.

#### Statistical analysis population

The modified intention-to-treat (mITT) analysis set includes all participants who have been randomized and have completed outcome measures for the baseline (T0) and at least one post-intervention session (T1, either after high-intensity stimulation or after low-intensity stimulation) without major protocol violations. Since mixed-effects models allow missing data, we will conduct all analyses using mixed-effects models on the mITT analysis set. All participants who were randomized but not part of the mITT analysis set will be described. However, since the aim is to investigate the effects of high-intensity tVNS vs. low-intensity tVNS, it is crucial to have completed outcome measures for the baseline and at least one post-intervention session (high-intensity or low-intensity stimulation). We will impute missing values of covariate predictors to maximise the sample size for controlled analyses based on correlated covariates (e.g., body fat values based on BMI, sex, and age or single questionnaire scores).

Major protocol violations include:

- Changes in participant’s criteria that preclude safe use of tVNS.
- Insufficient (i.e., 0 minutes logged by the tVNS device) at-home stimulation during the intervention phases

Completion of the study is independent of interim analyses, and the study will continue until the planned sample size is reached. Interim analyses will use the mITT population but include only participants who have completed primary endpoint data at the time of the interim analysis. We record treatment expectations and blinding and will assess the extent to which the results depend on the treatment expectations.

#### Primary endpoints

The primary endpoints of this study are peripheral (circulating immune cells and cytokines) and central inflammation (DBSI), as well as motivation (effort invigoration and maintenance of the EAT and concurrent fMRI data). To evaluate stimulation-induced changes in high- vs. low-intensity tVNS, we will use linear mixed-effects models (LMEs) implemented in R using the ‘*lmerTest’* package [56], which are suitable for repeated measures. We will include covariates (all centered) such as BMI, age, sex, and stimulation order and perform model comparison (using anova(), *X*^2^ in R) to determine the final model structure. Primary outcomes are assessed at baseline and after each stimulation phase (high- and low-intensity; 14 d stimulation each). Stimulation-induced changes in primary outcomes will be compared between high- and low-intensity stimulation conditions.

#### Secondary and other prespecified endpoints

Secondary endpoints are tVNS-induced changes in mood (PANAS), the correlation of tVNS-induced changes in peripheral and central inflammation with tVNS-induced changes in motivation and mood, and whether inflammation is associated with motivational indices or mood indices (orthogonal to the effects of tVNS). Moreover, we will evaluate the broader impact of tVNS across behavioral and mood domains as secondary endpoints (wanting and exertion ratings, utility slopes, mood and motivation in daily life, anhedonia (SHAPS-D), depressive symptoms (BDI-II), and apathy (AMI)). Secondary endpoints will be analyzed using LMEs or robust regression models, as appropriate. For LMEs the same covariates and predictors as described in the section primary endpoints will be used. Secondary outcomes are assessed at baseline and after each stimulation phase. For some secondary outcomes, there is a second baseline measurement before the second stimulation phase. In these cases, we will adjust the model accordingly. Stimulation-induced changes in secondary outcomes will again be compared between high- and low-intensity stimulation.

Other prespecified endpoints are tVNS-induced changes in metabolic blood parameters (triglyceride-glucose-index, HOMA-Index, triglycerides, HDL, LDL, HbA1c, adiponectin, leptin, (acyl-)ghrelin, and GLP-1), body measures (BMI, waist-to-hip ratio, resting heart rate, blood pressure, and percentage of body fat), physical activity, and in eating behavior including decision weights in the food choice task. In addition, we investigate tVNS-induced changes in state ratings in daily life collected via EMA and tVNS-induced changes in self-reported interoception. Stimulation-induced changes in other prespecified outcomes will again be compared between high- and low-intensity stimulation. We systematically assess side effects and will investigate potential predictors of tVNS efficacy.

#### Statistical threshold and software

All analyses and tabulations will be performed using MATLAB or R [73, 74]. Generally, we consider α < 0.05 as significant. For exploratory analyses as well as post hoc tests, we will account for multiple testing using the false discovery rate (FDR).

## Discussion

Overweight and obesity are associated with chronic low-grade inflammation. Since VNS has recently been approved for the treatment of an inflammatory condition, it is pivotal to evaluate if tVNS could be used to reduce inflammation as well. Studies to date have yielded inconsistent results with regard to chronic inflammation [7, 70, 85, 86]. This might be attributable to small sample sizes [85, 86], a lack of comprehensive inflammatory phenotyping [7], or variable application sites and stimulation settings [70]. Here, we evaluate an extended use (≥ 30 min of daily conventional stimulation over 14 d) in chronic inflammation. We use a single-blind, randomized crossover protocol with a high-intensity stimulation at the cymba conchae and a low-intensity stimulation as control condition in 60 participants with overweight/obesity and investigate tVNS-induced changes in a comprehensive panel of peripheral and central inflammatory markers, as well as in motivation and mood.

While the study protocol provides a framework to assess tVNS-induced mid-term effects on chronic low-grade inflammation, it holds several limitations. First, the use of high- vs. low-intensity stimulation comes with limitations in blinding. The common lab-based alternative to stimulate the earlobe as a control condition is not suitable for at-home treatments. However, this risk is mitigated by instructing the participants that we are testing two stimulation protocols, which they either feel or not feel. In addition, we assess participants’ belief of whether the vagus nerve was activated or not. Second, we may recruit participants with a higher willingness to change their weight, potentially confounding our outcomes. This risk is reduced by restricting prior weight change to 10% of their body mass. Third, in our study design, participants are instructed to stimulate the vagus nerve at home. This might limit compliance and adherence to the intervention. However, the tVNS device logs the use, so we can evaluate adherence and potential dose-related effects. In addition, a completion bonus and daily reminders via phone or email during the intervention phase, might help to increase participants’ compliance. Crucially, the safe use of tVNS devices at home is an advantage of the technique and our design deliberately captures a more naturalistic use scenario.

The proposed standardized conventional tVNS protocol with at least 30 min of daily stimulation for ~ 14-days may lead to greater benefits on chronic low-grade inflammation compared to previous non-invasive VNS study protocols relying on shorter stimulation times. In addition, the multimodal study design integrates multiple potentially inter-dependent outcomes, such as inflammation (central and peripheral), motivation (behavioral and neural response), and mood. For the first time, our study assesses whether extended use of tVNS reduces central inflammation using novel DWI-based proxies in humans. At a mechanistic level, the study could provide pathophysiological insights into the link between obesity and symptoms of depression, such as motivational and mood changes, via inflammatory processes. Consequently, stimulation of the vagus nerve may complement the current set of therapies if successful, including during a weight maintenance phase after initial weight loss due to other interventions (i.e., GLP-1 RA). More broadly, chronic low-grade inflammation associated with overweight and obesity might be a suitable model to evaluate potential anti-inflammatory effects of VNS-based interventions, which could facilitate future research to provide conclusive evidence on the potential of non-invasive VNS to treat chronic inflammatory conditions.

## Supplementary Information

Supplementary Material 1.

## Data Availability

No datasets were generated or analysed during the current study.

## Abbreviations

Abbreviation: Definition
AMI: Apathy Motivation Index
AP: Anterior-posterior
BDI-II: Beck Depression Inventory II
BMI: Body mass index
BSA: Bovine serum albumin
CAIP: Cholinergic anti-inflammatory pathway
CD: Cluster of differentiation
cD: Conventional dendritic cells
DBSI: Diffusion basis spectrum imaging
DEGS: German Health Interview and Examination Survey for Adults
DFG: German Research Foundation
DPBS: Dulbecco’s Phosphate Buffered Saline
DWI: Diffusion weighted imaging
EAT: Effort allocation task
EDTA: Ethylenediaminetetraacetic acid
ELISA: Enzyme-linked immunosorbent assay
EMA: Ecological momentary assessment
EPI: Echo-planar-imaging
FcR: Fc receptor
FDA: Food and Drug Administration
FDR: False discovery rate
fMRI: Functional magnetic resonance imaging
FSL: FMRIB Software Library
GLP-1: Glucagon-like Peptide-1
HbA1c: Hemoglobin A1C
HDL: High-density lipoprotein
HOMA: Homeostasis Model Assessment
IL: Interleukin
LDL: Low-density lipoprotein
LME: Linear mixed-effects model
mITT: Modified intention-to-treat
NULISA: Nucleic Acid Linked Immuno-Sandwich Assay
PA: Posterior-anterior
PANAS: Positive and Negative Affect Schedule
PBMC: Peripheral blood mononuclear cells
pDC: Plasmacytoid dendritic cells
RA: Receptor agonist
SHAPS-D: Snaith-Hamilton Pleasure Scale (German version)
SI: Supporting Information
SPM: Statistical Parametric Mapping
T1w: T1 weighted
Tconv: Conventional T-cells
TE: Echo time
TEMRA: Terminal effector memory re-expressing CD45RA
Th: T helper
TR: Repetition time
TNF: Tumor necrosis factor
Treg: Regulatory T cells
tVNS: Transcutaneous vagus nerve stimulation
VAS: Visual analog scale
