# Supplementary material for: tVNS and obesity-related inflammation: a prospective sham-controlled randomized crossover study protocol
Source: BMC Psychol. 2026 Aug 4;14:1133. doi: 10.1186/s40359-026-05219-5 (PMC13435442; doi:10.1186/s40359-026-05219-5)
Supplement: Supplementary file 1 [file 40359_2026_5219_MOESM1_ESM.docx]

# Supporting Information

# tVNS and obesity-related inflammation: a prospective sham-controlled randomized crossover study protocol

Luisa Kaluza^1,5^, Anne Kühnel^1,3^, Ernesto Picòn-Galindo^6^, Mona Hergenröther^1^, Hesham Alkhateeb^1^, Simon Michalski^1^, Dalila Juliana Silva Ribeiro ^6^, Susanne V. Schmidt^7^, Dagmar Wachten^6^, & Nils B. Kroemer^1-4*^

1 Section of Medical Psychology, Department of Psychiatry and Psychotherapy, University Hospital Bonn, University of Bonn, Bonn, Germany

^2^	Department of Psychiatry and Psychotherapy, Tübingen Center for Mental Health, University of Tübingen, Tübingen, Germany

^3^	German Center for Mental Health (DZPG), partner site Tübingen

^4^	German Center for Diabetes Research (DZD), Neuherberg, Germany

^5^	Department of Neuroimmunology and Neuromuscular Diseases, Centre for Neurology, University Hospital Bonn, University of Bonn, Bonn, Germany

^6^	Institute of Innate Immunity, Biophysical Imaging, University Hospital Bonn, University of Bonn, Bonn, Germany

^7.^	Institute of Clinical Chemistry and Clinical Pharmacology, University Hospital Bonn, 53127, Bonn, Germany.

**Corresponding author***

Prof. Dr. Nils B. Kroemer, [nkroemer@uni-bonn.de](mailto:nkroemer@uni-bonn.de)

Venusberg Campus 1, 53127 Bonn, Germany

**SI1. Antibodies used in flow cytometry**

**T cell panel 1**

| **Antibody reactivity** | **Fluochrome** | **Source** | **Catalog number** |
| --- | --- | --- | --- |
| CD3 | FITC | BioLegend | 300306 |
| CD4 | PerCP/Cy5.5 | BioLegend | 300530 |
| CD127 | APC | eBioscience | 17-1278-42 (Ref) |
| HLA-DR, DP, DQ | APC/Fire^750^ | BioLegend | 361712 |
| CD45RA | BV421 | BioLegend | 304130 |
| CD8 | BV510 | BioLegend | 344732 |
| CD45 | BV711 | BioLegend | 304050 |
| CD25 | PE | BioLegend | 302606 |
| CCR7 | PECy7 | BioLegend | 353226 |

**T cell panel 2**

| **Antibody reactivity** | **Fluochrome** | **Source** | **Catalog number** |
| --- | --- | --- | --- |
| CD3 | FITC | BioLegend | 300306 |
| CD4 | PerCP/Cy5.5 | BioLegend | 300530 |
| CD45RA | APC | BioLegend | 304112 |
| CCR6 | APC/Cy7 | BioLegend | 353432 |
| CXCR3 | BV421 | BioLegend | 353716 |
| CD8 | BV510 | BioLegend | 344732 |
| CD45 | BV711 | BioLegend | 304050 |
| CCR4 | PECy7 | BioLegend | 359410 |

**Mono/DC panel**

| **Antibody reactivity** | **Fluochrome** | **Source** | **Catalog number** |
| --- | --- | --- | --- |
| HLA-DR, DP, DQ | FITC | BioLegend | 361706 |
| CD88 | PerCP/Cy5.5 | BioLegend | 344312 |
| CD3 | APC | BioLegend | 300412 |
| CD19 | APC | BioLegend | 302212 |
| CD20 | APC | BioLegend | 302310 |
| CD56 | APC | BioLegend | 362504 |
| CD1c | APC/Fire750 | BioLegend | 331546 |
| CD16 | PB | BioLegend | 302032 |
| CD14 | BV510 | BioLegend | 301842 |
| CD45 | BV711 | BioLegend | 304050 |
| CD123 | PE | BioLegend | 396604 |
| CD141 | PECy7 | BioLegend | 344110 |

**SI2. Gating strategies**

**T-cell panel 1**

**T-cell panel 1:** gated on singlets, CD45^+^ cells. Tconv = conventional T-cells, Treg = regulatory T-cells, CM = central memory, EM = effector memory, TEMRA = terminal effector memory re-expressing CD45RA. Regulatory T-cells are further differentiated into activated and resting.

**T-cell Panel 2**

**T-cell panel 2:** gated on singlets, CD45^+^, CD3^+^, CD4^+^. Th = T-helper cells.

**Monocytes and dendritic cells**

**Monocytes and dendritic cells:** gated on singlets, CD45^+^. DC = dendritic cells, non-cMo = non-classical (including non-classcial and intermediate) monocytes, cMo = classical monocytes, ncMo = non-classical monocytes, iMo = intermediate Monocytes, cDC = conventional dendritic cells, pDC = plasmacytoid dendritic cells.
